# CNS embryonal tumour with concomitant novel *BRD4::CTRC1* fusion and *BCOR* internal tandem duplication – evidence for synergism and non-mutually exclusive alterations in CNS embryonal tumours

**DOI:** 10.1186/s40478-024-01741-y

**Published:** 2024-02-26

**Authors:** Sze Jet Aw, Enrica Ee Kar Tan, Sharon Yin Yee Low, Chik Hong Kuick, Jian Yuan Goh, Kenneth Tou En Chang

**Affiliations:** 1 Department of Pathology and Laboratory Medicine, KK Women’s and Children’s Hospital, 100 Bukit Timah Road, Singapore, 229899 Singapore; 2 Paediatric Haematology/Oncology Service, KK Women’s and Children’s Hospital, 100 Bukit Timah Road, Singapore, 229899 Singapore; 3 Neurosurgical Service, KK Women’s and Children’s Hospital, 100 Bukit Timah Road, Singapore, 229899 Singapore

**Keywords:** Central nervous system embryonal tumours, BRD4-LEUTX fusion, BCOR internal tandem duplication, DNA methylation profiling

Central nervous system (CNS) embryonal tumours Not Elsewhere Classified/Not Otherwise Specified (NEC/NOS) is a category of CNS embryonal tumours lacking genetic alterations of a defined classification group. Recently, Lebrun et al. described a patient with a CNS embryonal tumour with a *BRD4::LEUTX* fusion, which matched the methylation profile of ‘CNS embryonal tumour with *BRD4::LEUTX* fusion’ using the Heidelberg brain classifier v12.8 [1]. This follows the first case of a CNS embryonal tumour with the *BRD4::LEUTX* gene fusion described by Wong et al. [2]. In this report, we describe a 1-year-old girl with a CNS embryonal tumour that had a methylation profile matching to the same group. However, in our case, *BRD4* was fused to *CTRC1* and in addition, there was a concomitant *BCOR* internal tandem duplication (ITD) (see Fig. 1).

**Fig. 1:**
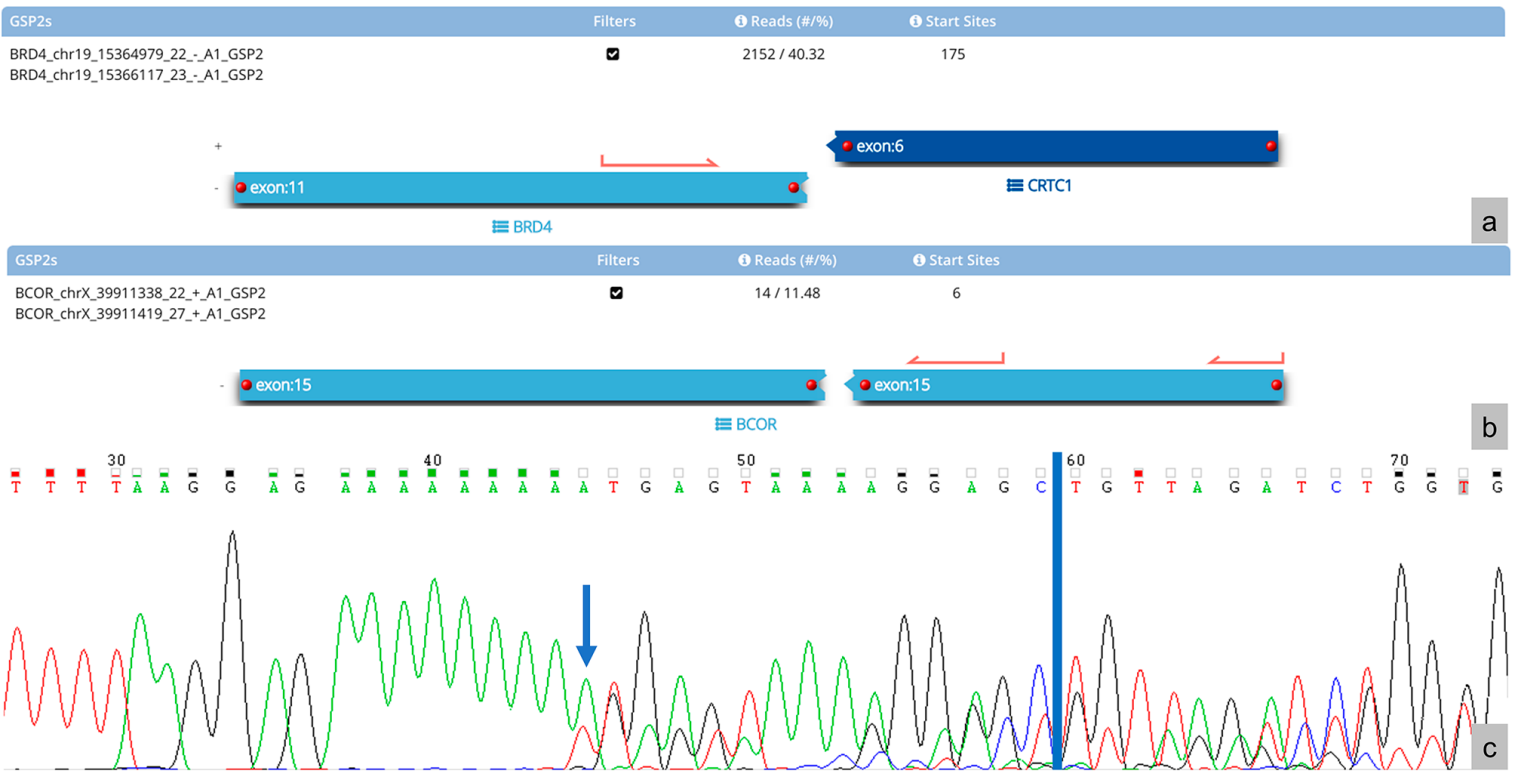
Sequencing results. **(a)***BRD4::CTRC1* transcript (40.32%) and **(b)***BCOR* ITD transcript (11.48%) with the respective percentage of unique reads spanning the breakpoint and supporting the event. **(c)** Electropherogram indicating the presence of the ITD breakpoint (located at the blue line). The overlapping peaks are due to the presence of two ITD transcripts, where one lacks an adenosine (‘A’) nucleotide (indicated by the arrow)

Clinically, the patient presented with left-sided weakness. Radiologically, there was a 5.4 cm right fronto-temporal lobe intra-axial brain tumour that enhanced heterogeneously. There were small cystic components and some calcifications.

Excision of the tumour showed predominantly large nests of monomorphic cells bearing minimal cytoplasm with large, hyperchromatic, irregular nuclei. Some areas show a trabeculated arrangement and some areas show cells featuring clear to eosinophilic cytoplasm (see Fig. 2). Notable immunohistochemical findings are diffuse positivity for synaptophysin, OLIG2, and BCOR, and loss of H3K27me3. Ki67 stained more than 90% of the cells. The high nuclear-to-cytoplasmic ratio of most tumour cells, positivity for synaptophysin and loss of H3K27me3 bore similarities to the tumour described by Lebrun et. al; however, the trabeculated arrangement, clear to eosinophilic cytoplasm, OLIG2 and BCOR positivity, and high Ki67 proliferative index were unique to our patient’s tumour [1]. Ampliseq Childhood Cancer Panel, a next-generation sequencing-based targeted gene panel, showed no reportable single nucleotide variants or copy number variants. Archer® Pan Solid Tumour v2 NGS panel, a high-throughput sequencing technique that identifies gene translocations and internal tandem duplications in solid tumours, showed the presence of a *BRD4::CRTC1* fusion (40.32% of unique reads), and a *BCOR* ITD (11.48% of unique reads). The *BCOR* ITD was identified to be within exon 15 and the duplicated segment was 415 base pairs (bp) long. Polymerase chain reaction and Sanger sequencing of the ITD breakpoint using primers: forward-5’-CACATGCTTTGGGATACGTTTGT-3’ and reverse-5’-AATTTCGTTCGTGAATTC-3’ confirmed the presence of the breakpoint. Interestingly, two ITD transcripts were detected, where one lacked an adenosine (‘A’) nucleotide (see Fig. 1c). DNA methylation analysis with the Heidelberg brain classifier v12.8 placed the tumour within the category of ‘CNS embryonal tumour with *BRD4::LEUTX* fusion’ (calibrated score: 0.98) [3]. Unlike Lebrun et. al’s case, the copy number variation profile did not show any significant chromosomal gains or losses.

The patient was treated with intensive chemotherapy as per Headstart II protocol. Due to fungal ventriculitis and viral reactivations, high dose chemotherapy with autologous transplant was postponed and two months of metronomic chemotherapy was given to bridge her cancer treatment while she was treated with antifungal and antiviral therapies. She subsequently underwent autologous transplant which was complicated by poor bone marrow recovery with viral reactivations. She underwent a successful haploidentical transplant four months later with good bone marrow recovery. Currently she is well and has been in remission for 1 year 11 months from end-of-treatment and 3 years 2 months from diagnosis.

**Fig. 2:**
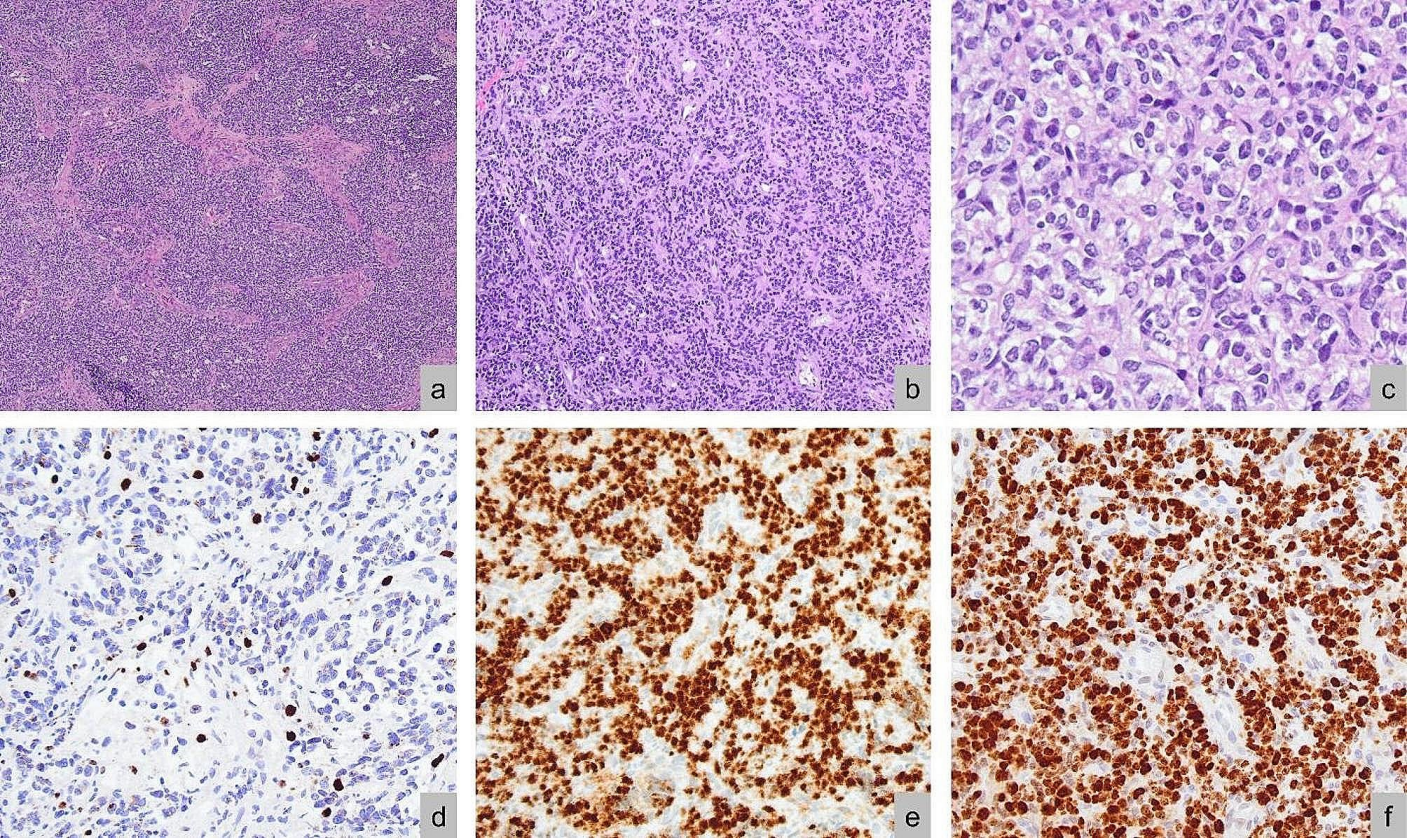
Histopathological features. **(a)** Tumour cells arranged in large nests (HE, magnification 40x). **(b)** Trabecular arrangement (HE, magnification 200x). **(c)** Tumour cells with ample cytoplasm (HE, magnification 400x). **(d)** Tumour cells showing H3K27me3 loss with retained expression in normal elements (magnification 200x) **(e)** Tumour cells showing diffuse and strong BCOR positivity with absent staining in normal elements (magnification 200x). **(f)** Ki67 index of the tumour cells is markedly elevated (> 90%) (magnification 200x). HE, hematoxylin-eosin

This case contributes a third *BRD4-*rearranged CNS embryonal tumour with a novel *BRD4::CRTC1* gene fusion. Noteworthy is the presence of *BCOR* ITD in a smaller percentage of sequencing reads of the tumour. CNS embryonal tumor with *BCOR* ITD is a separate category, and the significance of the presence of the *BCOR* ITD in our case is not clear. The presence of diffuse BCOR immunoreactivity and the methylation result in our tumour makes the possibility of a collision tumour (a *BRD4::CTRC1* tumour and a *BCOR*-ITD tumour) less likely. More likely, we hypothesize that the *BCOR-*ITD occurs concomitantly in at least a proportion of the *BRD4::CTRC1* tumour cells, as suggested by the lower transcript levels. Co-occurring mutations are well-described in cancer, especially when the alterations converge along complementary pathways with resultant synergistic effect on tumourigenesis [4]. Since BRD4 and BCOR do converge along several pathways (such as their interactions with polycomb repressive complexes and histone modifications), it is tenable that they can co-occur and synergistically impel tumourigenesis as dual oncogenic drivers [5, 6]. This may, in part, account for the unusually high Ki67 index we observe in our patient’s tumour (surpassing the rate reported by Lebrun et al. and CNS tumours with *BCOR* ITD, in general) [1].

In summary, we report a tumour with a novel *BRD4-CTRC1* gene fusion and concomitant *BCOR*-ITD which had a methylation profile of a ‘CNS embryonal tumour with *BRD4-LEUTX* fusion’. Identification of this novel fusion adds to the group of *BRD4-*rearranged tumours, particularly in the CNS. The novel gene partner *CTRC1* raises the consideration of renaming the aforementioned methylation category to ‘CNS embryonal tumour with *BRD4*-rearrangment’ [7]. Intriguingly, our tumour also has a concomitant *BCOR* ITD, suggesting that the molecular alterations in CNS embryonal tumours may not be mutually exclusive.

## Data Availability

Data sharing is not applicable to this article as no datasets were generated or analysed during the current study.
